# Running-app engagement and perceived training gains: sequential indirect associations through motivational affordances and runner motivation

**DOI:** 10.3389/fpsyg.2026.1989444

**Published:** 2026-10-28

**Authors:** Jenan Fathma Rande, January Naga, Ryan Ebardo

**Affiliations:** 1 College of Computer Studies, MSU-Iligan Institute of Technology, Iligan, Philippines; 2 College of Information and Computing Sciences, Mindanao State University, Marawi, Philippines; 3 College of Computer Studies, De La Salle University, Manila, Philippines

**Keywords:** exercise motivation, fitness applications, mHealth, motivational affordances, perceived performance, running, self-determinination theory, sport psychology

## Abstract

**Objective:**

Running applications provide feedback, goal setting, rewards, and social interaction, yet app use is often discussed as though it directly improves objective performance. This study tested a sport psychology model linking running-app engagement to perceived training gains via perceived motivational affordances and runner motivation.

**Methods:**

A quantitative, cross-sectional online survey was completed by 206 adult running-app users. Twenty-five five-point indicators were analyzed using partial least squares structural equation modeling with 5,000 bootstrap resamples. A two-step assessment examined the measurement model and then the structural model, including direct, specific indirect, and total effects. Common-method considerations, construct-level cross-validated prediction, and three sensitivity analyses were also evaluated.

**Results:**

Engagement was positively associated with motivational affordances (β = 0.707), motivation (β = 0.401), and perceived training gains (β = 0.401). Motivational affordances were positively associated with motivation (β = 0.496), and motivation was positively associated with perceived training gains (β = 0.499); all 95% bootstrap confidence intervals excluded zero. The indirect effect of engagement on perceived training gains through motivation was β = 0.200, while the sequential engagement–affordances–motivation–gains indirect effect was β = 0.175. The model explained 68.8% of the variance in motivation and 71.1% of the variance in perceived training gains.

**Conclusion:**

Runners who integrated an app more strongly into their training also reported greater perceived motivational value, motivation, and training gains. Positive statistical indirect associations linked engagement to perceived training gains via runner motivation and the sequentially specified affordance–motivation route. Given the cross-sectional design and reliance on self-reported perceived gains, these findings support an associational rather than causal interpretation.

## 1 Introduction

Mobile running applications have shifted everyday training from an activity merely performed to one that is continuously recorded, reviewed, shared, and compared. Pace traces, route maps, accumulated distance, goal prompts, badges, and social feedback can make training progress visible at a scale and frequency unavailable to most recreational runners a decade ago. Systematic reviews indicate that mobile and wearable interventions can increase physical activity, although effects are heterogeneous and often modest (Direito et al., 2017; Romeo et al., 2019; Mazeas et al., 2022). The central sports-science question is therefore not simply whether an application is present, but how engagement with its functions relates to the motivational processes through which runners sustain training and interpret progress.

Self-determination theory (SDT) provides a useful interpretive framework for considering how digital environments may support or frustrate autonomy, competence, and relatedness (Deci and Ryan, 2000; Teixeira et al., 2012). Running applications may support autonomy through self-selected goals, competence through immediate feedback and longitudinal progress displays, and relatedness through clubs, sharing, and encouragement. They may also undermine these experiences when feedback becomes controlling or social comparison produces pressure. Qualitative evidence from runners using Strava illustrates this dual character: participants describe motivation and connection alongside self-presentation concerns and social pressure (Couture, 2021). In the present study, however, the runner-motivation measure is a broad index and does not distinguish autonomous motivation, controlled motivation, or amotivation. SDT is therefore used to inform the interpretation of app-related motivational experiences, not as a direct test of the SDT regulation continuum.

An affordance describes an action possibility that emerges in the relationship between a user and a technology. Motivational affordances are design properties perceived as supporting goals, competence, social interaction, recognition, or enjoyment (Zhang, 2008). Tracking, analytics, personalized plans, goal setting, achievements, sharing, and comparison can all serve as motivational affordances when runners find them useful for training. Research on gamified services and fitness platforms suggests that feedback, achievement, and social features can foster engagement, but the extent of engagement depends on users' goals and context (Deterding et al., 2011; Hamari and Koivisto, 2015; Koivisto and Hamari, 2019). In a running-specific study grounded in SDT, social-exercise-platform functions strengthened motivation to participate in a sporting event (Tsai et al., 2021). Longitudinal Strava data further indicate that receiving social recognition can precede increases in running frequency, although observational platform effects remain vulnerable to selection and reciprocal influence (Franken et al., 2023).

Three gaps motivate the present study. First, prior research has provided important insights into the determinants of fitness-app use, motivation-related app features, and physical activity intentions (Yang and Koenigstorfer, 2021; Villalobos-Zúñiga and Cherubini, 2020). Further investigation is needed to clarify the distinct associations among running-app engagement, perceived motivational affordances, runner motivation, and running-related outcomes. Second, the present study assesses perceived training gains rather than objectively recorded running performance. This distinction is important because research comparing runners' self-reported data with GPS fitness-watch records found good reliability for weekly running distance but moderate reliability for running pace (Bullock et al., 2024). Third, cross-sectional path models can be used to examine theoretically specified indirect associations, but their findings do not establish temporal ordering or causal mediation (Rohrer et al., 2022; Maxwell et al., 2011; Maxwell and Cole, 2007). Accordingly, the present study distinguishes running-app engagement, perceived motivational affordances, runner motivation, and perceived training gains; examines their direct and sequential indirect associations; and interprets the findings in a manner consistent with the cross-sectional design.

The study asks whether runners who integrate an application more deeply into their routine perceive its features as more motivational, whether these affordances are associated with stronger motivation, and whether motivation is associated with perceived improvement in pace, distance, frequency, and training effectiveness. This contribution is a focused sports-technology model that integrates a concept from information systems, known as motivational affordance, with an established sport and exercise framework called SDT, while preserving the distinction between perceived gains and verified performance. Because the motivation measure does not distinguish forms of behavioral regulation, the model does not test whether autonomous, controlled, or amotivated regulation explains the observed associations.

## 2 Conceptual framework

### 2.1 Running-app engagement and motivational affordances

Running-app engagement in this study refers to the degree to which an application is embedded in training: runners use it during sessions, depend on it to monitor progress, inspect post-run statistics, and regard it as part of their routine. Repeated engagement increases exposure to and familiarity with tracking, analytics, goal, reward, social, and coaching functions. From an affordance perspective, these functions matter when users perceive them as enabling valued actions (Zhang, 2008). A runner who frequently reviews pace and training history has more opportunities to experience data as competence feedback; a runner who uses clubs and sharing can experience relatedness or comparison. Engagement should therefore be associated with the perceived motivational value of the feature set.

*H1. Running-app engagement is positively associated with perceived motivational affordances*.

### 2.2 Engagement, motivational affordances, and runner motivation

Engagement can be directly associated with motivation because routine self-monitoring makes goals salient, supplies feedback, and structures training. At the same time, use alone is not psychologically equivalent to useful use. SDT predicts that environments are motivational when they support autonomy, competence, and relatedness (Deci and Ryan, 2000; Teixeira et al., 2012). Goal setting and personalized plans can support autonomy; pace and distance feedback can support competence; and social sharing can support relatedness. Gamification may add challenge and recognition, but externally controlled rewards can be less beneficial than features that runners interpret as informational and self-relevant (Deterding et al., 2011; Hamari and Koivisto, 2015; Koivisto and Hamari, 2019). These propositions motivate the distinction between engagement and perceived motivational affordances. They do not imply that the present broad motivation index measures satisfaction of the three needs or a particular SDT regulation.

H2. Running-app engagement is positively associated with runner motivation.
H3. Perceived motivational affordances are positively associated with runner motivation.

Recent sports research provides three aligned precedents. Among marathon participants, flow statistically mediated associations involving event quality, self-efficacy, perceived value, and perceived performance (Wu et al., 2026). In fitness-application research, flow was also linked to application use and exercise behavior, with autonomous motivation conditioning the pathway (Song et al., 2026). In a Philippine physical education context, positive affect and willingness to reengage were more relevant to engagement than the utility and attainment dimensions of individual interest (Lobo, 2026). Together, these findings support examining the psychological processes that accompany participation in technology or sport, rather than treating exposure itself as sufficient.

### 2.3 Runner motivation and perceived training gains

Autonomous motivation is consistently associated with exercise participation and persistence (Teixeira et al., 2012). In running, motivated athletes are more likely to set goals, attend sessions, tolerate training discomfort, and persist long enough for adaptation to occur. The present outcome is deliberately labeled perceived training gains because respondents evaluated whether their pace, distance capacity, training frequency, overall performance, and training effectiveness had improved since using an application. These ratings may reflect genuine change, but without timed trials or longitudinal app logs, they cannot verify physiological or competitive performance.

H4. Runner motivation is positively associated with perceived training gains.

Engagement may also retain a direct association with perceived gains. Recording sessions and reviewing trends can improve training organization and provide actionable information even beyond generalized motivation. Accordingly, the model includes a direct path rather than assuming complete mediation.

H5. Running-app engagement is positively associated with perceived training gains.

### 2.4 Indirect pathways and hypotheses

The model specifies two complementary statistical indirect associations. First, engagement may be associated with perceived gains through runner motivation. Second, a more specific, sequentially specified route links engagement to perceived affordances, affordances to motivation, and motivation to perceived gains. Because all variables were measured concurrently, the ordering is theory-specified rather than temporally observed. Figure 1 summarizes the five direct hypotheses (H1–H5) and the two indirect-association hypotheses (H6–H7).

*H6. Running-app engagement has a positive indirect association with perceived training gains through runner motivation*.
*H7. Running-app engagement has a positive sequentially specified indirect association with perceived training gains through perceived motivational affordances and runner motivation*.

**Figure 1:**
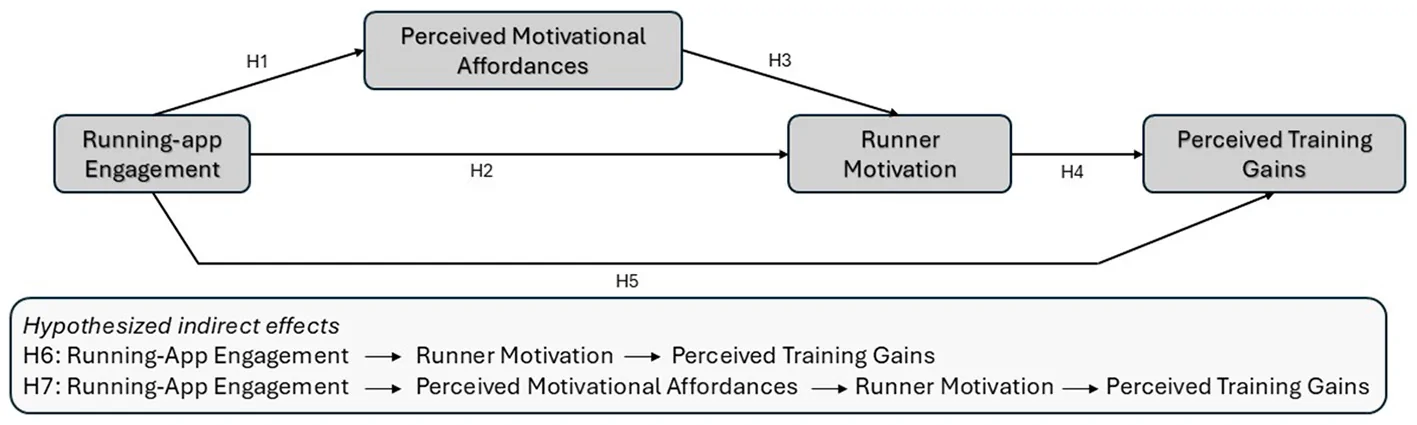
Proposed conceptual framework illustrating the hypothesized direct relationships **(H1–H5)** and indirect effects **(H6–H7)** among running-app engagement, perceived motivational affordances, runner motivation, and perceived training gains.

## 3 Methodology

### 3.1 Research design and participants

This was a cross-sectional online survey of adult running enthusiasts who used a mobile fitness application for training. A nonprobability convenience and purposive recruitment strategy was used through running communities, social media groups, fitness networks, and university or local networks. Eligibility required participants to be at least 18 years old, engage in running, and use a running application. The survey was open from 2 April to 17 June 2026 (77 days).

The analytic sample comprised 206 submitted responses. No item-level missing values were present in the 25 indicators used in the primary model. The sample size permits detection of an incremental effect of approximately *f*^2^ = 0.047 at 80% power and α = 0.05 for a regression with two predictors; this sensitivity calculation is reported instead of the heuristic ‘10-times rule,' which is not a reliable power analysis (Kock and Hadaya, 2018). Because recruitment was non-probability-based, statistical power does not eliminate selection bias or establish population representativeness.

### 3.2 Data collection and ethical considerations

Before accessing the questionnaire, participants viewed an information statement describing the study's purpose, expected completion time, the voluntary nature of participation, confidentiality provisions, and their right to discontinue participation before submitting their responses. Participants indicated electronic informed consent by selecting “I agree,” confirming that they were at least 18 years old and voluntarily agreed to participate. The University Ethics Review Board (UERB) approved the study protocol (UERB Code: UERB-2026-00157). Reporting was guided by the STROBE statement and relevant CHERRIES recommendations for web-based surveys (Von Elm et al., 2007; Eysenbach, 2004).

### 3.3 Instrumentation

All indicators were rated on a five-point scale ranging from 1 (strongly disagree) to 5 (strongly agree). The questionnaire contained researcher-developed and context-adapted items informed by prior literature on technology engagement, motivational affordances, exercise motivation, goal setting, gamification, and mobile physical-activity applications. The items were not administered as unmodified versions of established instruments.

Item development began by defining the conceptual domain of each construct. Candidate items were informed by the Sport Motivation Scale II, the Behavioral Regulation in Exercise Questionnaire, and research on motivational affordances, gamification, goal setting, technology use, and mobile physical-activity applications (Zhang, 2008; Deterding et al., 2011; Hamari and Koivisto, 2015; Markland and Tobin, 2004; Koivisto and Hamari, 2019; Pelletier et al., 2013). The indicators are described as literature-informed, context-adapted items rather than direct reproductions of the cited instruments. Supplementary Table S1 presents the complete wording of all indicators included in the primary model.

Before the main survey, a two-member expert panel with interdisciplinary research expertise in information technology and social sciences reviewed the questionnaire. The panel examined each candidate item for relevance, clarity, representativeness, and suitability for the running-app setting. The review resulted in simpler wording, the removal or consolidation of overlapping statements, clearer alignment between indicators and their intended constructs, and a distinction between routine app engagement, app-feature appraisals, and perceived training gains. The review was based on narrative comments and recommendations rather than numerical content-validity ratings.

Following expert review, the questionnaire was pilot-tested with 35 eligible running-app users who were not included in the main analytical sample. This pilot sample was sufficient to evaluate item clarity, questionnaire administration, response-scale suitability, and preliminary internal consistency. It was not used to establish definitive construct validity. The complete measurement model was subsequently evaluated using the main study sample of 206 respondents. Preliminary internal-consistency estimates were satisfactory: Cronbach's alpha was 0.932 for running-app engagement, 0.893 for perceived motivational affordances, 0.954 for runner motivation, and 0.919 for perceived training gains. Because these estimates were based on a relatively small pilot sample, they were interpreted as preliminary evidence and were reassessed in the main sample.

Running-app engagement was measured using five items assessing the extent to which a running application was integrated into the respondent's training routine. The items covered regular activity tracking, use of the application during most training sessions, reliance on the application for monitoring progress, post-training review of running statistics, and the application's importance within the training routine.

Perceived motivational affordances were measured using seven items assessing whether common application functions were experienced as motivationally useful. The items covered activity tracking, pace and distance feedback, goal setting, achievements or badges, social sharing, comparison with other runners, and personalized training guidance. The construct represents an aggregate appraisal of the motivational usefulness of the application's feature set rather than the presence or effect of any single function. It should not be interpreted as demonstrating that these functionally different features are interchangeable or equally motivational.

*Runner motivation* was measured using eight items covering enjoyment, satisfaction from improvement, physical and mental wellbeing, personal challenge, fitness goals, progress tracking, health and fitness motives, and encouragement from in-app achievements. Collectively, the items form a broad runner-motivation index. The measure is not an unmodified SDT instrument and does not distinguish autonomous motivation, controlled motivation, and amotivation. It therefore cannot identify which form of behavioral regulation accounts for the observed associations.

*Perceived training gains* were measured using five items assessing perceived improvements in running pace, distance capacity, running frequency, overall performance, and training effectiveness. These outcomes represent respondents' perceptions rather than objectively recorded changes in running performance. An additional item concerning consistent goal achievement was included only in the alternative six-item sensitivity analysis.

The expert review and the 35-participant pilot provided preliminary evidence concerning item clarity, content coverage, questionnaire administration, and score consistency. Evidence from the main sample was subsequently examined through indicator loadings, reliability coefficients, AVE, and HTMT. These complementary procedures provide preliminary measurement evidence for the present exploratory analysis but do not fully establish definitive construct validity, particularly for the broad runner-motivation index and the heterogeneous motivational-affordance indicators.

### 3.4 Data analysis

Partial least squares structural equation modeling (PLS-SEM) was selected because the study emphasized exploratory prediction, estimation of a specified network of direct and indirect associations, and assessment of construct-level predictive relevance using context-adapted measures (Hair et al., 2021). Covariance-based SEM would have been more appropriate if the principal objective had been strict confirmation of an established measurement structure through global covariance-model fit. The prespecified model was estimated with 5,000 case-resampled bootstrap samples and repeated cross-validation. Running-app engagement, runner motivation, and perceived training gains were specified in Mode A because their indicators were treated as correlated manifestations of broader construct-level tendencies. Perceived motivational affordances were likewise specified in Mode A to represent a generalized appraisal of the motivational usefulness of the application's feature set. This operationalization concerns the shared appraisal across functions rather than the independent contribution of tracking, goals, badges, social sharing, comparison, or guidance. Given the heterogeneity of these functions, the resulting construct should be interpreted as an exploratory aggregate appraisal; formative, composite, and feature-specific alternatives remain plausible measurement specifications.

The measurement model was evaluated using indicator loadings, Cronbach's alpha, composite reliability (CR), average variance extracted (AVE), and heterotrait–monotrait ratios (HTMT), with discriminant validity assessed using the HTMT criterion (Henseler et al., 2015). Indicators with loadings between 0.40 and 0.70 were retained when their removal was not required to achieve acceptable CR or AVE and would reduce the construct's theoretical content coverage (Hair et al., 2021). The structural model was assessed using inner variance inflation factors (VIFs), coefficients of determination (*R*^2^ and adjusted *R*^2^), Cohen's f^2^ effect sizes, and construct-level cross-validated *Q*^2^ values. The Q^2^ assessment used 10 repetitions of 10-fold cross-validation and was treated as a supplementary predictive check rather than as an indicator-level PLSpredict [19].

Three robustness and sensitivity analyses were conducted. First, the model was re-estimated after excluding 17 participants who selected the same response option across all 25 primary-model indicators. These uniform-response cases were retained in the primary analysis because uniform responding alone was not considered sufficient evidence of invalidity. Second, the model was re-estimated using an alternative six-item specification of perceived training gains. Third, an outcome regression was estimated after adjusting for age, gender, running experience, running frequency, app-use frequency, weekly running distance, and duration of app use, with HC3 heteroskedasticity-consistent standard errors. Potential common-method variance was examined using an unrotated single-factor diagnostic. This procedure was treated as a preliminary diagnostic rather than as definitive evidence for or against common-method bias (Podsakoff et al., 2003). All statistical tests were two-sided, with α = 0.05.

## 4 Results

### 4.1 Participant and app-use characteristics

Participants were predominantly aged 18–34 years (83.5%) and male (73.3%). Approximately two-thirds had one year or less of running experience (66.5%), and 68.0% ran one to three times per week. Overall, 72.3% used a running application during every run (56.3%) or most runs (16.0%). Among the 201 respondents who identified the applications they used, 137 (68.2%) selected Strava, frequently alongside another platform. Table 1 summarizes the participant and running app profiles.

**Table 1:** Participant and running-app profile (*N* = 206).

Characteristic	Category	*n*	%
Age	18–24 years	96	46.6
	25–34 years	76	36.9
	35–44 years	32	15.5
	45 years and above	2	1.0
Gender	Male	151	73.3
	Female	55	26.7
Running experience	Less than 6 months	78	37.9
	6 months−1 year	59	28.6
	1–3 years	51	24.8
	More than 3 years	18	8.7
Running frequency	Once per week	56	27.2
	2–3 times per week	84	40.8
	4–5 times per week	51	24.8
	Daily	15	7.3
App-use frequency	Every run	116	56.3
	Most runs	33	16.0
	Sometimes	39	18.9
	Rarely	18	8.7
Weekly distance	< 5 km	62	30.1
	5–10 km	69	33.5
	10–20 km	31	15.0
	>20 km	44	21.4

Percentages use the full analytical sample (*N* = 206) and may not sum to 100.0% because of rounding.

### 4.2 Common-method considerations

All focal constructs were measured concurrently using the same online questionnaire. In the unrotated single-factor diagnostic, the first factor accounted for 59.3% of total indicator variance, exceeding the commonly applied 50% heuristic. This diagnostic does not quantify the extent of common-method bias or establish that the structural associations are spurious; however, it indicates substantial shared variance and means that common-method concerns cannot be dismissed. The path coefficients and explained variance are therefore interpreted cautiously as potentially inflated same-source associations.

### 4.3 Measurement model

The Mode A measurement model was evaluated using indicator loadings, Cronbach's alpha, composite reliability (CR), average variance extracted (AVE), and heterotrait-monotrait ratios (HTMT). As reported in Table 2, alpha ranged from 0.889 to 0.953, CR from 0.915 to 0.961, and AVE from 0.608 to 0.786, while indicator loadings ranged from 0.610 to 0.920. Social sharing (FAF5; loading = 0.677) and comparison with others (FAF6; loading = 0.610) were retained to preserve coverage of the social domain; their lower loadings nevertheless indicate greater heterogeneity within the affordance appraisal. HTMT values ranged from 0.743 to 0.847 (Table 3), below the 0.90 criterion. Thus, the scores demonstrated acceptable internal consistency and empirical distinction in the present sample. These results concern the internal measurement structure and do not, by themselves, resolve the theoretical breadth of runner motivation or the semantic proximity of engagement, affordance appraisal, motivation, and perceived gains.

**Table 2:** Construct descriptives, reliability, and convergent validity.

Construct	k	M	SD	α	CR	AVE	Loading range
Engagement	5	4.07	0.96	0.932	0.948	0.786	0.864–0.912
Affordances	7	3.98	0.84	0.889	0.915	0.608	0.610–0.848
Motivation	8	4.30	0.83	0.953	0.961	0.756	0.806–0.916
Gains	5	4.04	0.85	0.916	0.939	0.757	0.757–0.920

k = number of indicators; M = mean; SD = standard deviation; α = Cronbach's alpha; CR = composite reliability; AVE = average variance extracted. Means were calculated from item scores ranging from 1 to 5.

**Table 3:** Heterotrait-monotrait ratios.

Construct	Running-app engagement	Perceived motivational affordances	Runner motivation	Perceived training gains
Engagement	—	—	—	—
Affordances	0.743	—	—	—
Motivation	0.796	0.803	—	—
Gains	0.833	0.824	0.847	—

### 4.4 Structural model and hypothesis testing

Before evaluating the hypothesized relationships, structural collinearity was assessed using inner variance inflation factors. The maximum VIF was 2.00 for the runner-motivation equation and 2.30 for the perceived-training-gains equation. These values were below conventional thresholds, indicating that structural collinearity was not problematic (Hair et al., 2021). All five direct-path estimates were positive, and their 95% bootstrap confidence intervals excluded zero. Running-app engagement was positively associated with perceived motivational affordances (H1: β = 0.707, *p* < 0.001). When engagement and affordances were entered simultaneously as predictors of runner motivation, both engagement (H2: β = 0.401, *p* < 0.001) and affordances (H3: β = 0.496, *p* < 0.001) were positively associated with motivation. Runner motivation was positively associated with perceived training gains (H4: β = 0.499, *p* < 0.001), while running-app engagement retained a positive direct association with perceived training gains (H5: β = 0.401, *p* < 0.001). Accordingly, H1-H5 were supported as associational hypotheses; complete bootstrap estimates and hypothesis decisions are reported in Table 4.

**Table 4:** Direct structural effects and hypothesis decisions.

Hypothesis, decision, and path	β	Boot SE	*T*	*P*	95% CI	*f* ^2^
H1 (supported): Engagement → Affordances	0.707	0.048	14.729	< 0.001	[0.603, 0.793]	1.002
H2 (supported): Engagement → Motivation	0.401	0.071	5.648	< 0.001	[0.257, 0.535]	0.258
H3 (supported): Affordances → Motivation	0.496	0.064	7.750	< 0.001	[0.368, 0.621]	0.394
H4 (supported): Motivation → Gains	0.499	0.056	8.911	< 0.001	[0.386, 0.609]	0.375
H5 (supported): Engagement → Gains	0.401	0.063	6.365	< 0.001	[0.273, 0.520]	0.242

β = standardized path coefficient; Boot SE = bootstrap standard error; CI = percentile bootstrap confidence interval; *f*^2^ = effect size. Confidence intervals were obtained from 5,000 bootstrap samples. Conventional *f*^2^ values of approximately 0.02, 0.15, and 0.35 indicate small, medium, and large effects, respectively.

The model explained 50.1% of the variance in perceived motivational affordances, 68.8% in runner motivation, and 71.1% in perceived training gains. As reported in Table 5, repeated 10-fold cross-validation produced positive construct-level *Q*^2^ values ranging from 0.491 to 0.705, indicating lower out-of-sample prediction error than the corresponding training-fold mean benchmark. Figure 2 presents the estimated direct paths and explained variance for the endogenous constructs.

**Table 5:** Explanatory and construct-level predictive performance.

Endogenous construct	*R* ^2^	Adjusted *R*^2^	Cross-validated *Q*^2^
Perceived motivational affordances	0.501	0.498	0.491
Runner motivation	0.688	0.685	0.673
Perceived training gains	0.711	0.709	0.705

*Q*^2^ was calculated using 10 repetitions of 10-fold cross-validation on construct scores.

**Figure 2:**
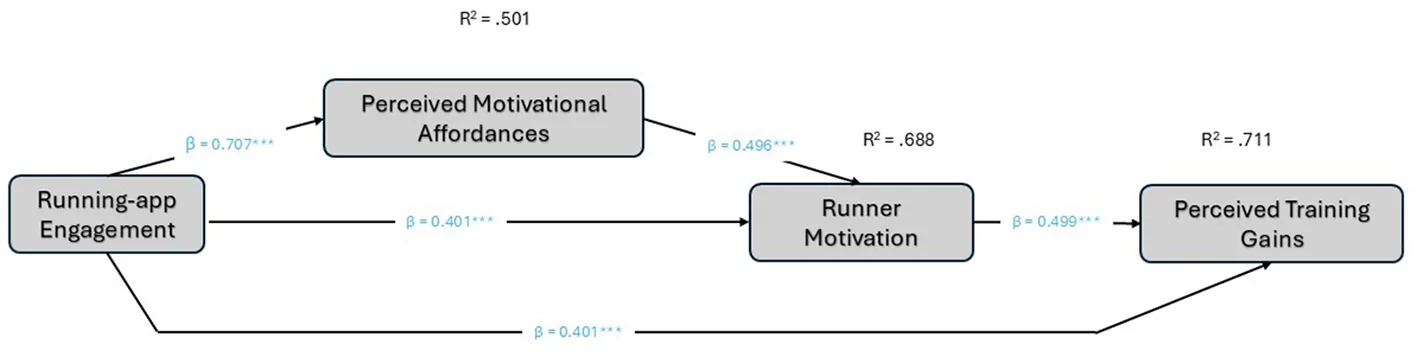
Estimated structural model showing standardized path coefficients (β), statistical significance, and explained variance (R^2^) of the endogenous constructs.

### 4.5 Indirect effects

Both hypothesized indirect associations were statistically supported. Running-app engagement had a positive indirect association with perceived training gains through runner motivation (H6: β = 0.200, 95% CI [0.126, 0.281], *p* < 0.001). The sequentially specified indirect association through perceived motivational affordances and runner motivation was also positive (H7: β = 0.175, 95% CI [0.108, 0.252], *p* < 0.001). These pathways produced a total indirect association of β = 0.376. The direct engagement–gains association remained positive after inclusion of the indirect routes, yielding concurrent direct and indirect associations in the specified model. Because all constructs were measured at the same time, these effects decompose contemporaneous covariance and do not demonstrate temporal or causal mediation. Table 6 reports the specific indirect, total indirect, and total effects.

**Table 6:** Indirect and total effects.

Hypothesis, decision, and effect	β	Boot SE	*T*	*P*	95% CI
H6 (supported): Engagement → Motivation → Gains	0.200	0.039	5.128	< 0.001	[0.126, 0.281]
H7 (supported): Engagement → Affordances → Motivation → Gains	0.175	0.037	4.730	< 0.001	[0.108, 0.252]
Total indirect, Engagement → Gains	0.376	0.048	7.833	< 0.001	[0.282, 0.470]
Affordances → Motivation → Gains	0.248	0.047	5.277	< 0.001	[0.159, 0.346]
Total effect, Engagement → Gains	0.777	0.041	18.951	< 0.001	[0.688, 0.848]

β = standardized effect; Boot SE = bootstrap standard error; CI = percentile bootstrap confidence interval. Confidence intervals were obtained from 5,000 bootstrap samples. The total indirect effect was calculated using unrounded coefficients and may therefore differ slightly from the sum of the displayed rounded specific indirect effects.

### 4.6 Robustness and sensitivity analyses

Several supplementary analyses were conducted to determine whether the primary results were sensitive to response-pattern screening, outcome specification, and observed participant characteristics. First, 17 participants provided uniform responses across all 25 indicators included in the primary model. Because uniform responding does not necessarily indicate an invalid response, these cases were retained in the primary analysis and excluded only in a sensitivity analysis. Their exclusion produced minimal changes in the standardized direct-path coefficients, with no absolute change exceeding 0.017. Second, the model was re-estimated using an alternative six-item specification of perceived training gains. The resulting coefficients were similar to those obtained from the primary five-item specification: the engagement-gains coefficient changed from 0.401 to 0.395, while the motivation-gains coefficient changed from 0.499 to 0.514. Finally, a controlled outcome regression was estimated using the complete sample (*N* = 206). Running-app engagement (*B* = 0.356, HC3 SE = 0.091, *p* < 0.001) and runner motivation (*B* = 0.479, HC3 SE = 0.070, *p* < 0.001) remained independently associated with perceived training gains after adjustment for age, gender, running experience, running frequency, app-use frequency, weekly running distance, and duration of app use. None of the demographic, running-related, or app-use control variables showed a statistically significant independent association with perceived training gains at the *p* < 0.05 level. Overall, the direction and magnitude of the focal associations remained stable across the supplementary specifications. As shown in Table 7, these analyses reduce concern that the primary findings depended on a particular item specification, response-pattern decision, or set of observed controls.

**Table 7:** Sensitivity of direct path coefficients.

Path	Primary model (*N* = 206)	Uniform-response cases excluded (*n* = 189)	Six-item gains specification (*N* = 206)
Engagement → Affordances	0.707	0.691	0.707
Engagement → Motivation	0.401	0.397	0.401
Affordances → Motivation	0.496	0.499	0.496
Engagement → Gains	0.401	0.388	0.395
Motivation → Gains	0.499	0.506	0.514

Values are standardized PLS path coefficients. Uniform-response cases were participants who selected the same response option across all 25 indicators.

## 5 Discussion

### 5.1 Principal findings

The findings are consistent with a layered associational model linking running-app engagement, app-feature appraisals, a broad runner-motivation index, and perceived training gains. At the same time, the constructs share content related to monitoring, feedback, progress, achievement, and improvement. Although the HTMT values suggest that the construct scores were not empirically identical, they do not rule out semantic overlap. Moreover, all variables were measured concurrently in the same questionnaire, and the first unrotated factor accounted for 59.3% of item variance. Shared wording, response consistency, and common-method variance may therefore have contributed to the strong coefficients and high *R*^2^ values. The estimated magnitudes should be interpreted as same-source associations rather than as precise estimates of temporally ordered psychological effects.

The affordances–motivation association was somewhat larger than the direct engagement–motivation association. This pattern is consistent with the proposition that exposure is insufficient: runners respond to what tracking, goals, rewards, coaching, and social features allow them to do. The strongest social-feature indicators loaded less strongly than tracking, analytic, goal, and achievement indicators. One interpretation is that the motivational value of sharing and comparison varies more across runners. This interpretation aligns with research showing that social fitness platforms can generate connection and encouragement for some users while creating pressure and self-presentation concerns for others (Franken et al., 2023; Russell et al., 2023).

Runner motivation and engagement both retained independent associations with perceived training gains. This pattern is compatible with SDT-informed evidence linking self-determined motivation to exercise participation and persistence (Teixeira et al., 2012), but the present broad index does not identify autonomous or controlled regulation and therefore does not directly test that SDT mechanism. The engagement association may reflect training organization, information, or awareness of improvement. It may also partly reflect same-source attribution: respondents who identify strongly as app users may be more inclined to credit the app for improvement. Because the outcome was framed relative to app use and was not verified against training logs, this alternative explanation cannot be excluded.

### 5.2 Theoretical contributions

The study contributes by connecting motivational-affordance reasoning with an SDT-informed perspective in a runner-specific model. Affordance reasoning explains why the same function may matter differently across users: a leaderboard can represent challenge and competence feedback to one runner but controlling comparison to another. SDT helps interpret the possible motivational quality of such experiences, but the present measures do not test psychological need satisfaction or the regulation continuum. Separating engagement from affordances nevertheless clarifies a recurring conceptual problem in fitness-technology research. Frequent use is behavioral integration; perceived affordance is an appraisal of what the technology enables; the broad motivation index captures heterogeneous running motives and experiences; and perceived training gain is an evaluative outcome.

The findings complement recent evidence that flow may connect marathon experiences with perceived performance (Wu et al., 2026), that autonomous motivation may shape psychological pathways between fitness-app use and exercise behavior (Song et al., 2026), and that affective attraction and willingness to re-engage are important aspects of individual interest in a Philippine sport context (Lobo, 2026). This literature suggests that simple exposure to an application may be less informative than the motivational experience accompanying its use.

### 5.3 Practical implications

At the practical level, the findings indicate that runners' overall appraisal of an application's motivational usefulness is associated with motivation and perceived training gains. The study did not isolate or experimentally compare individual app functions; it therefore provides no direct evidence that particular forms of ranking, notification, social comparison, personalized guidance, or autonomy-supportive feedback produce superior outcomes. Previous research nevertheless suggests potentially useful directions for design evaluation, including adjustable goals, interpretable feedback, user control over visibility and notifications, and configurable comparison features. These possibilities should be treated as design propositions for future testing rather than as effects demonstrated in the present study.

For coaches and recreational runners, application data are best treated as aids to reflection rather than proof of improvement. A dashboard can help organize training and sustain attention, but perceived improvement should be checked against objective indicators such as standardized time trials, pace at a given heart rate, completed volume, injury-free consistency, or race results. Coaches can also discuss whether feedback is informative or pressuring, especially when athletes show compulsive checking or anxiety around public activity records.

### 5.4 Limitations and future research

The study has several strengths, including its sport-specific sample, mechanism-focused model, complete data for the primary indicators, explicit bootstrap testing of two indirect pathways, and multiple sensitivity analyses. Nevertheless, several limitations qualify the findings. The cross-sectional design cannot establish directionality, temporal ordering, or causality; the indirect effects represent same-time-point statistical associations rather than causal mediation. All focal constructs were measured using the same questionnaire. The first unrotated factor accounted for 59.3% of total indicator variance, indicating substantial shared variance, although this diagnostic cannot determine the extent of common-method bias. Similar item framing, shared measurement context, and respondents' general evaluative tendencies may therefore have contributed to the path coefficients and *R*^2^ values.

The measures were researcher-developed or substantially adapted. Expert review and pilot testing provided preliminary support, but numerical content-validity indices were unavailable, and the main-sample reliability, AVE, and HTMT results do not establish comprehensive construct validity. The runner-motivation index combines enjoyment, health, challenge, goals, progress tracking, and app-related encouragement; high internal consistency does not establish that these elements represent a single SDT process. Similarly, the motivational-affordance indicators cover heterogeneous functions. Their Mode A specification represents an aggregate appraisal, while formative, composite, or feature-specific specifications may also be defensible. Semantic proximity among monitoring, feedback, progress, achievement, and perceived improvement may further contribute to the observed associations.

The convenience sample was predominantly 18–34 years old (83.5%), male (73.3%), and relatively inexperienced (66.5% had one year or less of running experience). The results therefore should not be generalized without qualification to women, older runners, experienced runners, competitive athletes, or runners who do not use applications. Perceived training gains were also self-reported rather than verified using application logs, wearable-device data, standardized tests, or race results.

Future measurement development should extend the initial expert review and pilot testing through larger multidisciplinary panels, documented quantitative content-validity assessment, cognitive interviews, and independent-sample confirmation. Runner motivation should be assessed using validated multidimensional instruments that distinguish autonomous motivation, controlled motivation, amotivation, and psychological need satisfaction. Alternative specifications should compare a general affordance-appraisal factor with formative, composite, and feature-specific models. Longitudinal studies should measure engagement, app-feature appraisals, motivation, and outcomes at appropriately separated time points and recruit more balanced samples by gender, age, experience, and competitive level. Where consent permits, survey responses should be linked with application or wearable-device records. Experimental and micro-randomized studies are also needed to estimate the effects of particular feedback, goal, reward, notification, comparison, and social features. Future studies should also reduce common-method risk through temporally separated measurement, multisource data, objective application records, and, where appropriate, theoretically unrelated marker variables or latent method-factor approaches.

## 6 Conclusion

Among 206 running-app users—predominantly young, male, and relatively inexperienced—greater integration of an application into the running routine was associated with stronger aggregate appraisals of motivational usefulness, a higher broad runner-motivation score, and greater perceived training gains. Positive statistical indirect associations were observed through runner motivation and through the sequentially specified motivational-affordance–motivation route. However, the proposed ordering remains a theoretical model requiring longitudinal confirmation. The findings concern perceived training gains and should not be interpreted as evidence of objectively improved running performance.

## Data Availability

The datasets presented in this article are not readily available because the dataset is not publicly available due to confidentiality and privacy considerations. Requests to access the datasets should be directed to january.naga@g.msuiit.edu.ph.
