# Supplementary material for: Running-app engagement and perceived training gains: sequential indirect associations through motivational affordances and runner motivation
Source: Front Psychol. 2026 Oct 28;17:1989444. doi: 10.3389/fpsyg.2026.1989444 (PMC13751230; doi:10.3389/fpsyg.2026.1989444)
Supplement: Supplementary file 1 [file Table_1.docx]

Supplementary Material

**Supplementary Table S1. Measurement items and primary-model indicator loadings.**

| **Code** | **Construct** | **Item wording** | **Loading** |
| --- | --- | --- | --- |
| FAU1 | Running-app engagement | I regularly use a fitness application to track my runs. | 0.890 |
| FAU2 |  | I use a running app during most of my training sessions. | 0.912 |
| FAU3 |  | I depend on running apps to monitor my training progress. | 0.864 |
| FAU4 |  | I check my running statistics through the app after training. | 0.882 |
| FAU5 |  | Fitness apps are an important part of my running routine. | 0.885 |
| FAF1 | Perceived motivational affordances | The activity tracking feature motivates me to run more. | 0.830 |
| FAF2 |  | Seeing my distance and pace statistics encourages me to improve. | 0.837 |
| FAF3 |  | Goal-setting features help me stay committed to running. | 0.845 |
| FAF4 |  | Achievements, badges, or rewards motivate me to continue running. | 0.848 |
| FAF5 |  | Social sharing features encourage me to run more often. | 0.677 |
| FAF6 |  | Comparing my performance with others motivates me to improve my running. | 0.610 |
| FAF7 |  | Personalized coaching or training plans help improve my running. | 0.775 |
| RM1 | Runner motivation | I run because I enjoy the experience of running. | 0.849 |
| RM2 |  | I feel satisfied when I improve my running performance. | 0.913 |
| RM3 |  | Running makes me feel good physically and mentally. | 0.916 |
| RM4 |  | I run because I enjoy challenging myself. | 0.887 |
| RM5 |  | I run to achieve personal fitness goals. | 0.806 |
| RM6 |  | I run because tracking my progress motivates me. | 0.834 |
| RM7 |  | I run because I want to improve my health and fitness. | 0.877 |
| RM8 |  | Seeing my achievements in the app encourages me to continue running. | 0.869 |
| RP1 | Perceived training gains | My running pace has improved since using fitness apps. | 0.881 |
| RP2 |  | I can run longer distances than before. | 0.891 |
| RP3 |  | I run more frequently because of the app. | 0.757 |
| RP4 |  | My overall running performance has improved. | 0.891 |
| RP5 |  | Fitness apps help me train more effectively. | 0.920 |

*Note.* Note. All items were rated on a five-point agreement scale. The primary perceived-training-gains construct comprised RP1–RP5. RP6 (“I achieve my running goals more consistently when using fitness apps”) was included only in the alternative six-item sensitivity analysis.
